# Novel features in the structure of P-glycoprotein (ABCB1) in the post-hydrolytic state as determined at 7.9 Å resolution

**DOI:** 10.1186/s12900-018-0098-z

**Published:** 2018-12-13

**Authors:** Nopnithi Thonghin, Richard F. Collins, Alessandro Barbieri, Talha Shafi, Alistair Siebert, Robert C. Ford

**Affiliations:** 1 School of Biology, Faculty of Biology Medicine and Health, Michael Smith Building, The University of Manchester, Oxford Road, Manchester, M13 9PL UK; 2 eBIC, Diamond Light Source Ltd, Diamond House, Harwell Science & Innovation Campus, Oxfordshire, Didcot OX11 0DE UK

**Keywords:** P-glycoprotein, ABCB1, ATP-binding cassette, Transporter, Membrane protein, Protein structure, Cryo-electron microscopy

## Abstract

**Background:**

P-glycoprotein (ABCB1) is an ATP-binding cassette transporter that plays an important role in the clearance of drugs and xenobiotics and is associated with multi-drug resistance in cancer. Although several P-glycoprotein structures are available, these are either at low resolution, or represent mutated and/or quiescent states of the protein.

**Results:**

In the post-hydrolytic state the structure of the wild-type protein has been resolved at about 8 Å resolution. The cytosolic nucleotide-binding domains (NBDs) are separated but ADP remains bound, especially at the first NBD. Gaps in the transmembrane domains (TMDs) that connect to an inner hydrophilic cavity are filled by density emerging from the annular detergent micelle. The NBD-TMD linker is partly resolved, being located between the NBDs and close to the Signature regions involved in cooperative NBD dimerization. This, and the gap-filling detergent suggest steric impediment to NBD dimerization in the post-hydrolytic state. Two central regions of density lie in two predicted drug-binding sites, implying that the protein may adventitiously bind hydrophobic substances even in the post-hydrolytic state. The previously unresolved N-terminal extension was observed, and the data suggests these 30 residues interact with the headgroup region of the lipid bilayer.

**Conclusion:**

The structural data imply that (i) a low basal ATPase activity is ensured by steric blockers of NBD dimerization and (ii) allocrite access to the central cavity may be structurally linked to NBD dimerization, giving insights into the mechanism of drug-stimulation of P-glycoprotein activity.

**Electronic supplementary material:**

The online version of this article (10.1186/s12900-018-0098-z) contains supplementary material, which is available to authorized users.

## Background

P-glcoprotein (P-gp/ABCB1) is a member of the ATP-binding cassette (ABC) family of proteins [1–3]. It operates as an ATP-dependent exporter in the plasma membrane and it exports a host of small, mostly hydrophobic, molecules from the cell. Its principal role is to protect against xenobiotic compounds entering the organism from the diet or environment. However in some cancers being treated by chemotherapy, the high mutation rate, combined with the strong selective pressure of the drug, frequently leads to up-regulation of P-gp and multi-drug resistance [4, 5]. There is therefore an unmet need for an effective and reversible P-gp inhibitor to address its mediation of multi-drug resistance in cancer. To this end, structure-informed drug design/optimisation may be useful.

There are now many atomic models and experimental density maps for P-gp deposited in the Protein Databank (PDB) and Electron Microscopy Databank (EMDB) [6–14], and data are available at a resolution allowing a direct modeling of the amino acid residue side-chains [6–13]. Murine P-gp has so far been predominant in these studies. The murine version of the protein favours an inward-facing conformation (i.e. where the transmembrane domains –TMDs- surround a cavity leading to the cytoplasm and with the nucleotide-binding domains –NBDs- separated). This configuration of the protein has been proposed to represent the higher affinity state for transported substrates (allocrites) such as drugs or xenobiotic compounds. Until recently, all high resolution P-gp structures were inward-facing and in the absence of nucleotide, a condition that is non-physiological. One inward-facing structure for P-gp does display nucleotide at NBD1, but this structure was obtained after removal of the central linker polypeptide that joins NBD1 and TMD2. This large deletion inactivated the protein which showed only basal ATPase activity. However a structure for P-gp in the nucleotide-bound, outward-facing state was determined recently. For this study, mutation of the glutamate residues involved in ATP hydrolysis was needed [6]. Apart from inactivating the protein, the mutagenesis (E- > Q) also removed two charges in negatively-charged patches which must approach each other in the ATP-bound NBD-NBD interface. The change in electrostatics may favour the formation of an NBD1-NBD2 dimer. In the outward-facing state the TMDs surround a cavity leading to the extracellular milieu and the NBDs are sandwiched together, concertedly binding two ATP molecules. Hence the structural and biochemical data so far could be interpreted as implying that wild-type, active P-gp is very dynamic and exists only transiently in the outward-facing state, even in the presence of high ATP concentrations [14, 15].

Biophysical measures of P-gp dynamics have similarly demonstrated that the inward-facing configuration was dominant, also in the presence of a non-hydrolysable ATP analogue [15, 16]. This suggests that prevention of ATP hydrolysis alone does not favour the outward-facing state, and was consistent with the idea that electrostatic repulsion between the NBDs was more important. However these studies also found that, when trapped in a post-hydrolytic state, P-gp existed in a mixture that favoured the outward-facing conformation [16]. We therefore decided to study wild-type murine P-gp under these conditions using cryo-electron microscopy and single particle analysis. Our hypothesis was that this would allow the study of the conformation(s) of the protein without mutagenesis and in a physiologically-relevant state with nucleotide present.

## Results

### Cryo-EM

**Fig. 1:**
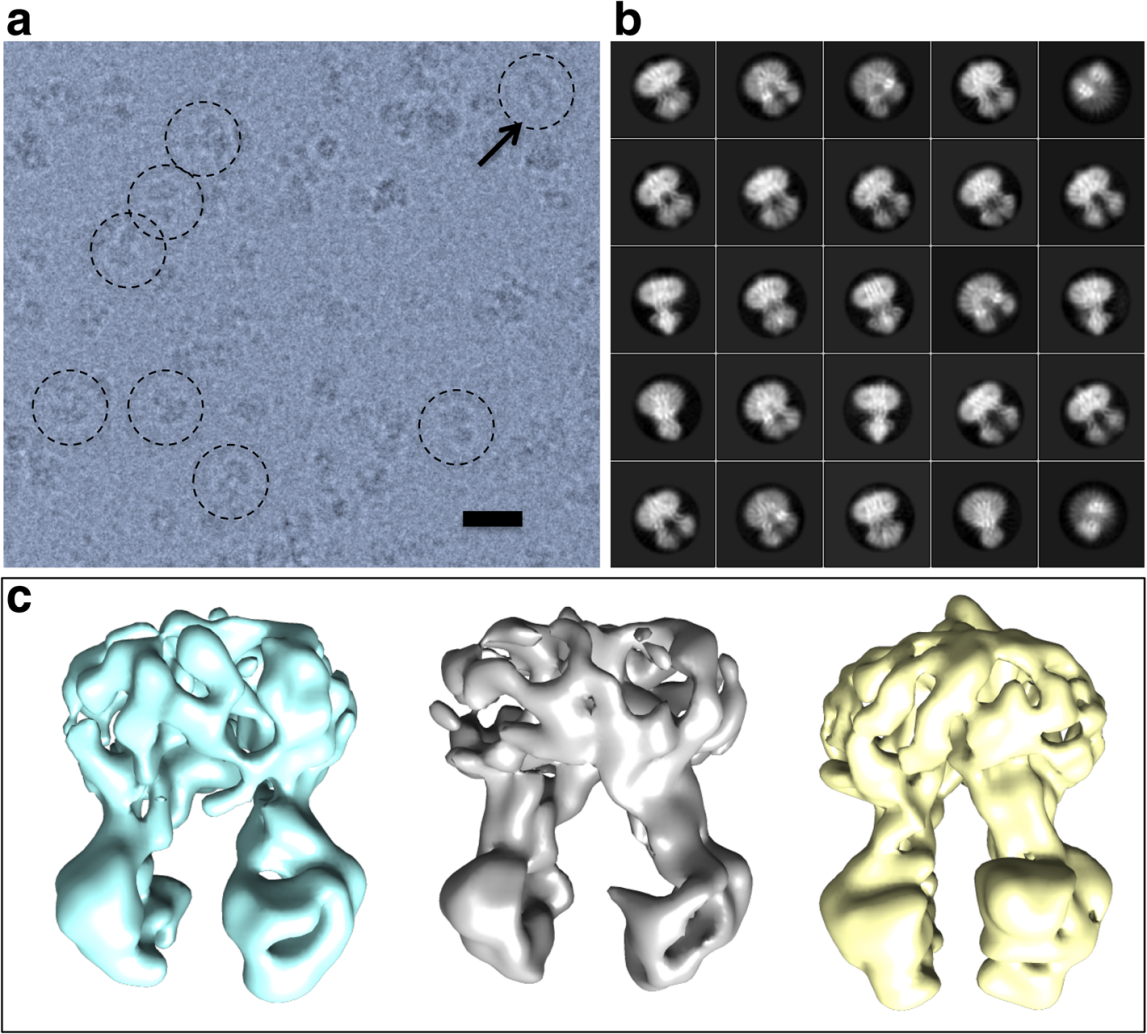
**a** Structural data. Example of a field of P-glycoprotein particles (darker grey) embedded in vitreous ice (lighter grey). Scale bar = 20 nm. The micrograph was filtered to 8 Å resolution for clarity. Various orientations of the particles in the ice gave rise to different projections. Encircled are examples of projections corresponding to side-on views of the molecule (i.e. perpendicular to the long axis), with a clear inward-facing conformation indicated (arrow). **b** Reference-free classification of the particle data set identified different projection classes and **c** 3D classification yielded 3 classes all corresponding to an inward-facing conformation of the protein. The class displayed in yellow was generated from the largest group of particles and this dataset was selected for further 3D refinement

Motion-corrected electron micrographs of the vanadate-trapped murine P-gp showed a homogeneous particle distribution across the electron microscopy grids, with few small aggregates (Fig. 1). This was consistent with the purification characteristics of the protein which displayed a symmetrical peak of mass about 250 kDa using size-exclusion chromatography (Additional file 1: Figure S1). Particles displayed a variety of projections; side-on projections could be readily discerned, showing a distinctive annular detergent micelle surrounding the TMDs at one end of the protein (Fig. 1a, circled particles). A flow chart of the image processing and 3D reconstruction strategy is shown in Additional file 1: Figure S2. After selecting particles, classification revealed well-differentiated 2D projection classes with internal features consistent with the resolution of helical secondary structural elements (Fig. 1b). Subsequent image processing of the entire particle dataset yielded three 3D classes that were very similar and all displayed an inward-facing conformation (Fig. 1c). The particle dataset corresponding to the 3D class with the highest resolution was split into two and each half was independently refined to allow for assessment of the resolution limitations of the structural analysis using the Fourier shell correlation (Additional file 1: Figure S3). A Fourier shell correlation value of 0.143 was reached at 7.9 Å. Local resolution of the 3D map was assessed using the Resmap algorithm, with only the detergent micelle showing significantly lower resolution than 8.6 Å (Additional file 1: Figure S3).

### Interpretation of the map

**Fig. 2:**
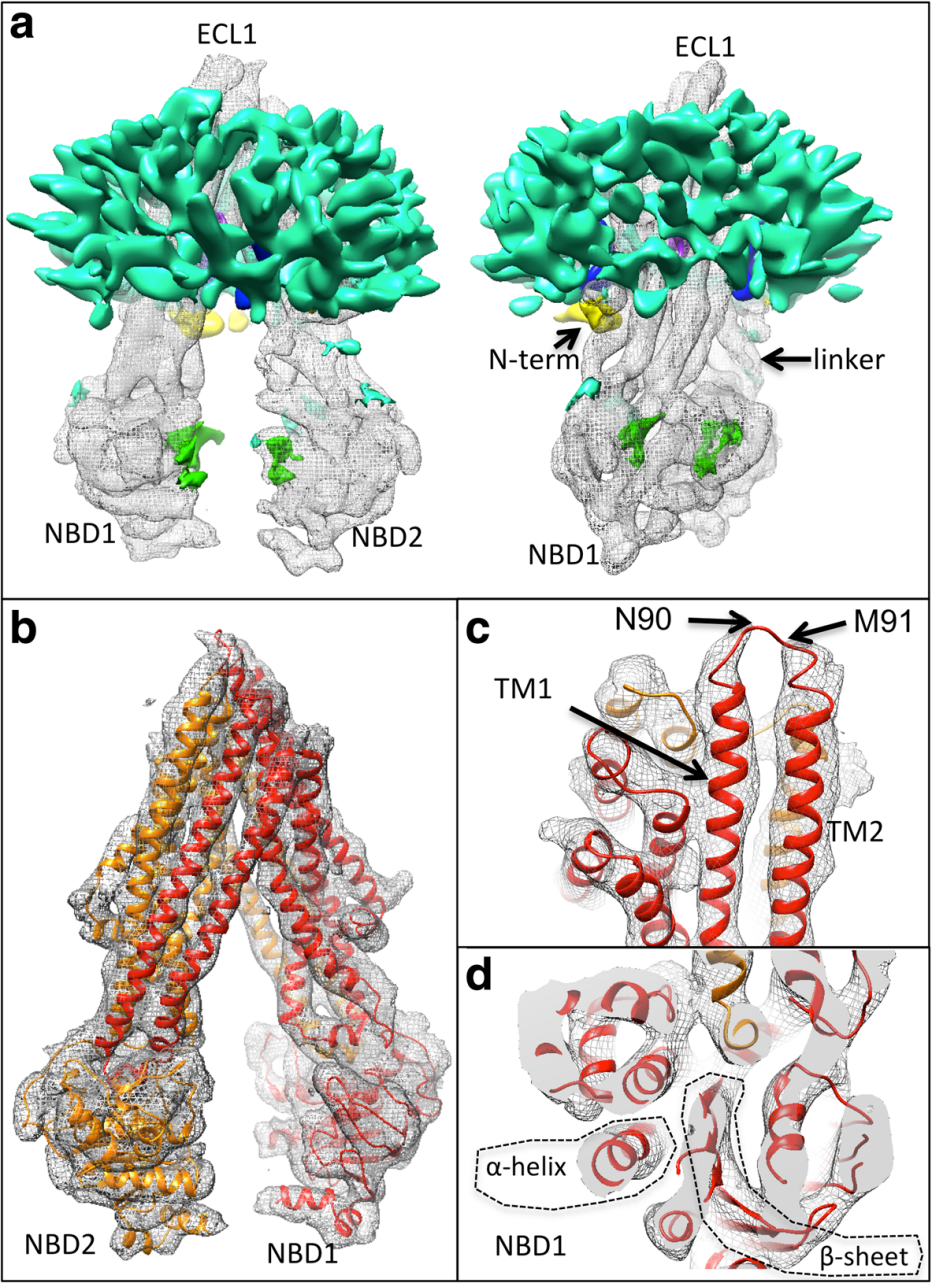
Cryo-EM map of P-glycoprotein derived from the highest resolution 3D class. **a** Orthogonal views of the map and different regions of the map are coloured according to their interpretation: Grey mesh – regions equivalent to the existing structures and within 2 Å of the fitted model (4ksb after MDFF); Turquoise – detergent micelle and unassigned density; Yellow – N-terminal extension (background/left); Green – putative ADP molecules; Purple and Blue – inner features associated with drug binding (mostly obscured in this panel) . The map is displayed at a threshold enclosing a volume of 75 × 10^3^ Å^3^ for the core regions (grey mesh) and 85 × 10^3^ Å^3^ for features not accounted for by the fitted model. **b** Core regions of the map with the MDFF-refined model shown as red and orange ribbon representations (N- and C-terminal halves of the protein, respectively). **c** Shows the extracellular portions of the map, with density lacking for 2 residues (N90, M91, arrows). **d** Shows part of NBD1. Individual β-strands viewed end-on cannot be resolved whilst individual α-helices in different orientations can be identified

Several previously-obtained mouse P-gp atomic models were tested using MDFF, with the inward-facing state corresponding to the PDB code *4ksb* having the best final cross-correlation coefficient with the experimental map [12]. MDFF using the 4ksb model yielded a global change in the rigid body-fitted model, with a narrowing of the angle between the TMDs and a shortening of the distance between the NBDs. Figure 2 shows the overall 3D density map using colours to indicate different features of the P-gp/detergent micelle complex that were interpreted after MDFF.

### Detergent (DDM) micelle (turquoise density)

This portion of the map in Fig. 2a appears as a hollow annular shell surrounding the hydrophobic membrane-spanning portions of P-gp. The maltoside polar headgroup region of the detergent molecules appears to form this density. An analysis of the micelle using the Resmap software [17] implies that the micelle is less ordered than the core regions of the protein (Additional file 1: Figure S3).

### P-gp core domains (grey mesh)

**Fig. 3:**
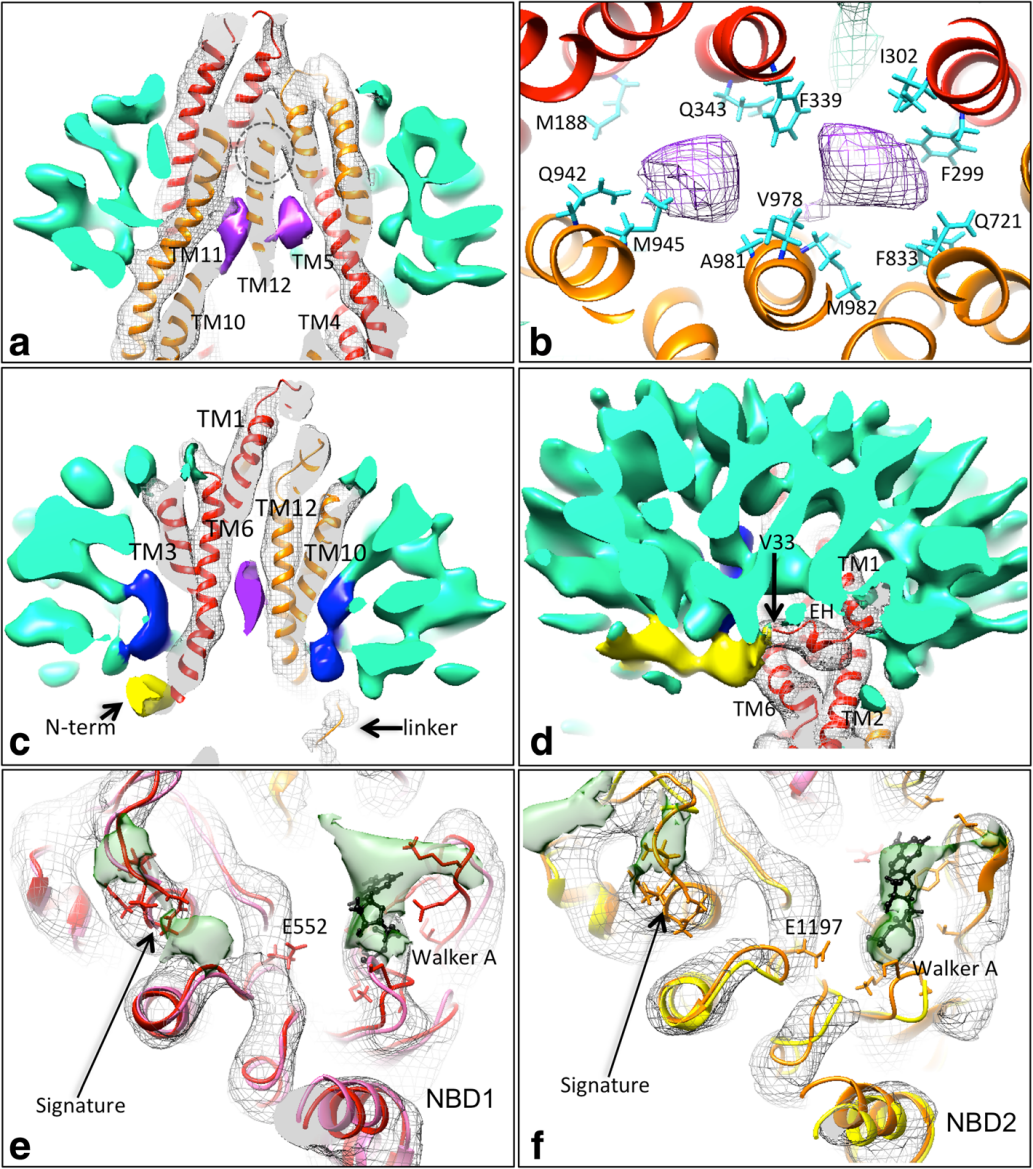
Novel features of the cryo-EM map: **a**,**b** Sections through the transmembrane region. **a** Shows two additional high density features (purple) that sit just below the cyclic peptide inhibitor binding site (dashed circle) and mostly formed by transmembrane (TM) helices 10,11,12,4,5 & 6 (clipped away for clarity). **b** Residues surrounding the two central densities (purple mesh) in the MDFF atomic model viewed from the extracellular side. **c** Blue density continuous with the detergent micelle (turquoise) fills the gaps formed by the inverted ‘V’ of TM helices 3&4 (left) and 9&10 (right,TM helices 4 and 9 are clipped away for clarity). **d** Additional density (yellow) below the detergent micelle (turquoise) that extends to the start of the elbow helix (EH) beginning at V33 in the fitted model (4ksb). This wraps over the protruding TM6 that connects to NBD1. **e**,**f** Additional densities at the interfacial surfaces of NBD1 and NBD2 (green transparent surface). Density for the core regions is shown in grey mesh. In both panels elongated additional densities are located at the expected ADP/vanadate site (right hand side in both panels). Two further small densities (left side in each panel) lie close to the Signature region. MDFF-refined models with ADP (red, orange traces) and without ADP (pink, yellow) are shown with few systematic differences except for the MDFF-refined ADP (black, ball and stick representation). The Signature sequence, Walker A residues and the conserved catalytic glutamates in the Walker B regions are highlighted with stick representation. Red/pink traces represent the N-terminal half of the molecule; orange/yellow the C-terminal half

Figure 2b shows the atomic model and the grey mesh highlights any regions of the map within 2 Å of the atoms of the MDFF-refined model. All transmembrane portions of the model except for the region around N90 & M91 in the first extracellular loop (Fig. 2c) lie within the experimental map. Individual α-helices in the NBDs are observed as discrete cylindrical entities, however, individual β–strands cannot be resolved in the β–sheet displayed in Fig. 2d. These observations are consistent with the estimation of the resolution of the overall 3D map and of local regions within the map. Densities in the map not consistent with the P-gp core domains and detergent micelle are described further in Fig. 3.

### Drug binding sites (purple density)

Initial structures for P-gp with cyclic peptide inhibitors [7, 8] showed an inhibitor binding site close to the apex of the internal V-shaped cavity of the TMDs. We observed no density in the equivalent region of the P-gp map (Fig. 3a, dashed circle). However immediately below this, towards the cytoplasmic side of the membrane, we observed two strong densities that were not accounted for by the initially-fitted model, nor by the MDFF-refined model. Figure 3b indicates the P-gp residues predicted to be surrounding these two densities in the map.

### TMD gaps (blue density)

ABC transporters in the same class as P-gp display gaps between the 4th and 6th membrane-spanning helices in each TMD when in the inward-facing conformation. These gaps will expose the inner leaflet of the phospholipid membrane to water that fills the internal cavity created by the teepee-shape of the transporter in this conformation. Here we observed L-shaped regions of density protruding into these gaps between TM helices 3 and 4 and between TM helices 9 and 10. These densities extend from the lower surface of the detergent micelle (Fig. 3c).

### N-terminal extension and linker region (yellow density)

A 30 Å-long extended density that joins with the N-terminal ‘elbow’ helix of P-gp can be observed in the map (Fig. 3d). This region is also clearly distinguishable from the micelle in the map before sharpening (Additional file 1: Figure S4) and has a finger-like shape. The fingertip merges with the headgroup region of the detergent micelle. In contrast, in TMD2 a 25 Å-long curved density connects with its elbow helix, and wraps around the surface of the cytoplasmic portion of TMD2 helix 12. This additional density fades out in the map as it extends between the NBDs in a location close to the start of TMD helix 9.

### Nucleotide binding (green density)

Two additional regions of density lie outside the NBDs in the expected positions for nucleotides. These are probably due to ADP and vanadate, and MDFF using the 4ksb model with ADP molecules included yielded a very similar result (Fig. 3e, f) but with some local rearrangements in the Walker A region. The density close to NBD1 (Fig. 3e) displays higher density than that at NBD2 (Fig. 3f).

We noted some weak additional density near the Signature regions and between the NBDs (Fig. 3e, f). The Signature region is characteristic of all ABC proteins and it must closely approach the ATP binding site formed by the Walker A and B regions in the opposing NBD during NBD dimerization. It seems possible that this additional density could represent some local organization of the linker region (see above). Although nearly all crystal structures of P-gp are without nucleotide, removal of the NBD1-TMD2 linker region did allow ATP to be resolved at NBD1 (PDBID 5koy, [9]) which is consistent with the idea that the linker region may modulate nucleotide binding sites.

## Discussions and conclusions

Visual inspection of the raw particle data did not allow unequivocal identification of P-gp in the outward-facing state, but particles in the inward-facing conformation with NBDs separated were unambiguously identifiable in the particle population. This was consistent with the 2D class averages which showed classes in the inward-facing state with NBDs separated, but no classes showing an obvious outward-facing separation of TMDs on the cytoplasmic side of the molecule. Given the signal:noise ratio of raw cryo-EM data it seems possible that the classification algorithms could have failed to distinguish some outward-facing state particles from (somewhat similar) projections of inward-facing state particles where the NBDs were roughly in line. For example in Fig. 1 some of the 2D classes shown might be consistent with the outward-facing conformation. However, subsequent 3D classification resulted in only inward-facing classes that were distinguished solely by the magnitude of the angle subtended between the TMDs. Similarly, forcing a 3D refinement with a low-resolution starting model representative of the outward-facing state nevertheless resulted in a refined structure that was clearly in the inward-facing state. Thus we concluded that the majority of the particles we studied were in the inward-facing state.

According to the resolution assessment via Fourier shell correlation, the structural data obtained was at *lower* resolution than was possible by cross-linking and extensive mutagenesis of the protein [6, 13]; however it was at *higher* resolution than data previously obtained by cryo-EM for active P-gp in the presence of a stabilising antibody fragment [14]. Flexibility may be a pre-requisite for active protein, hence may be an intrinsic limitation to structural studies of single particles where there are no constraints due to a crystal lattice.

The 3D density map clearly showed a well-defined detergent micelle region. The density is mainly formed by the maltoside headgroups making the micelle appear hollow. A similar phenomenon was reported in other cryo-EM studies of membrane proteins at resolutions exceeding 10 Å. This is probably because the hydrocarbon chains of the detergent molecules do not scatter electrons significantly more than the surrounding solvent (water). In a study examining the 3D structure of a bacterial inner membrane complex Wzz in DDM it was shown that the annular micelle could encase several small independent Wzz transmembrane domains [18]. The micelle surrounding P-glycoprotein does not show a smooth surface, but rather has a granular nature presumably as a result of local organization of the detergent polar headgroups due to the high curvature of the micelle. It is likely that water molecules intercalate into this region of the micelle and account for the lower density areas in the micelle [19].

P-gp core domains were well resolved and the MDFF-refined model was readily housed within the available densities with the only exception being in the region around the modeled positions of residues N90 and M91 in extracellular loop 1. This loop is normally glycosylated, with 3 consensus N-glycosylation sites, one being N90. In the *P. pastoris* expression system employed, only core glycosylation is likely to be present. In some prior structural studies, the three glycosylation sites were mutated to glutamine prior to crystallisation of the protein [7, 8] implying that this loop may be flexible, variable in its glycosylation and could hinder 3D crystal formation. Although cryo-EM studies of the inward-facing conformation of ABC family members have revealed that the NBDs may be less ordered than the TMDs [20, 21], in the current map, the NBDs are equally well resolved.

In terms of extra densities observed in the vicinity of transmembrane region, the origins of these densities remains enigmatic as there was no drug added in these structural studies. Detergent itself has been identified as a P-gp allocrite [22], hence the densities may represent the headgroup regions of two DDM molecules. Higher resolution data is needed to test this hypothesis. Other studies of P-glycoprotein have aimed to identify allocrite binding sites: The Zosuquidar-bound structure of a human/mouse P-glycoprotein chimera [13] showed two drug molecules bound at the same location as the two additional densities in Fig. 3b. Compared to the map presented in Fig. 3, the cavity for Zosuquidar appears to be slightly smaller – perhaps because of the cross-linking of the NBDs or the addition of the UIC2 antibody. These factors may have forced a narrower angle to be subtended between the two TMDs in the Zosuquidar study [13]. Similarly, a study of P-glycoprotein with cyclic peptides designed to probe the allocrite-binding pocket showed two molecules bound in the same locations as in Fig. 3. In contrast, a study of P-glycoprotein with a strong inhibitor showed its binding site to be asymmetric and much closer to the extracellular side of the molecule [10]. The binding site residues in Fig. 3b were also predicted in an *in-silico* molecular docking study of various P-gp allocrites [23]. Hence various structural studies suggest that the binding pocket is large and has the potential to be occupied by two allocrites simultaneously.

Densities protruding between TM helices 3 and 4 and between TM helices 9 and 10 appear to be due to distortion of the micelle in this region, with the hypothesis that maltoside headgroups kink inwards to fill the gaps that would otherwise lead to the exposure of detergent hydrocarbon chains to the aqueous central vestibule. It is possible that, in-vivo, inner lipid leaflet-located molecules may behave similarly, allowing access to the transporter via these two portals. In support of this interpretation, studies have shown that detergent molecules such as DDM can operate as P-gp substrates at low concentrations [22].

So far, structural data for the highly charged N-terminus of P-gp is still lacking. Herein, a kinked density at the corresponding position was revealed. If this density were to be fitted with 3 short α-helices with two breaks, it would account for most of the remaining ~ 30 residues of the P-gp N-terminus. Unambiguous assignment of the secondary structure in this region is stymied by the current resolution limitations, however. If this region in the protein behaves similarly in-vivo, then it will likely interact with the polar headgroup region of the plasma membrane and would have to move from this position in order to allow NBD dimerization and the formation of the outward-facing state. Similarly, little structural data exists for the P-gp linker region (residues 627–691) that joins the end of NBD1 with the elbow helix of TMD2 (residues 692–702). Here, a curved density corresponding to residues 684–691 of the 4ksb atomic model was discovered in a position that is juxtaposed between the two NBDs. This implies that the C-terminal end of the linker region is capable of being ordered under certain conditions, and (akin to CFTR/ABCC7 in the inward-facing conformation [20, 21]), may play a role in regulation of NBD dimerisation.

Densities corresponding to ADP molecules were displayed in the map. The density found close to NBD1 was significantly higher than the one at NBD2 implying that the NBD1 site may have a higher occupancy. Several reports suggest that NBD1 has a higher affinity for ATP and ADP/vanadate than its C-terminal counterpart [9, 24, 25].

The identification of significant regions in the map not accounted for by the atomic model was checked for model- and fitting-independence. This was done by comparing the results described above with those obtained by a very simple rigid body fitting of the first-half and second-half structural units of the atomic models. Even with this basic fitting procedure, all the above-mentioned additional density regions were apparent.

The current structural data implies that the ADP/vanadate state (post-hydrolytic state) can be predominantly inward-facing with the NBDs separated. This is somewhat different to conclusions from a recent electron paramagnetic resonance (EPR) study of spin-labeled cysteine residues that suggested that more than half P-gp molecules would be in the outward-facing state in the presence of vanadate and nucleotide [16]. A prior cryo-EM study of vanadate-trapped P-gp at low resolution (EMD-3427) also showed an inward-facing configuration [14], although with the caveat that the conformation was stabilized by a conformation-specific antibody. Nevertheless, the NBD separation and overall configuration of P-glycoprotein shows a close correlation between the current 8 Å-resolution map and the 20 Å-resolution map with F_AB_ bound (Additional file 1: Figure S4).

**Fig. 4:**
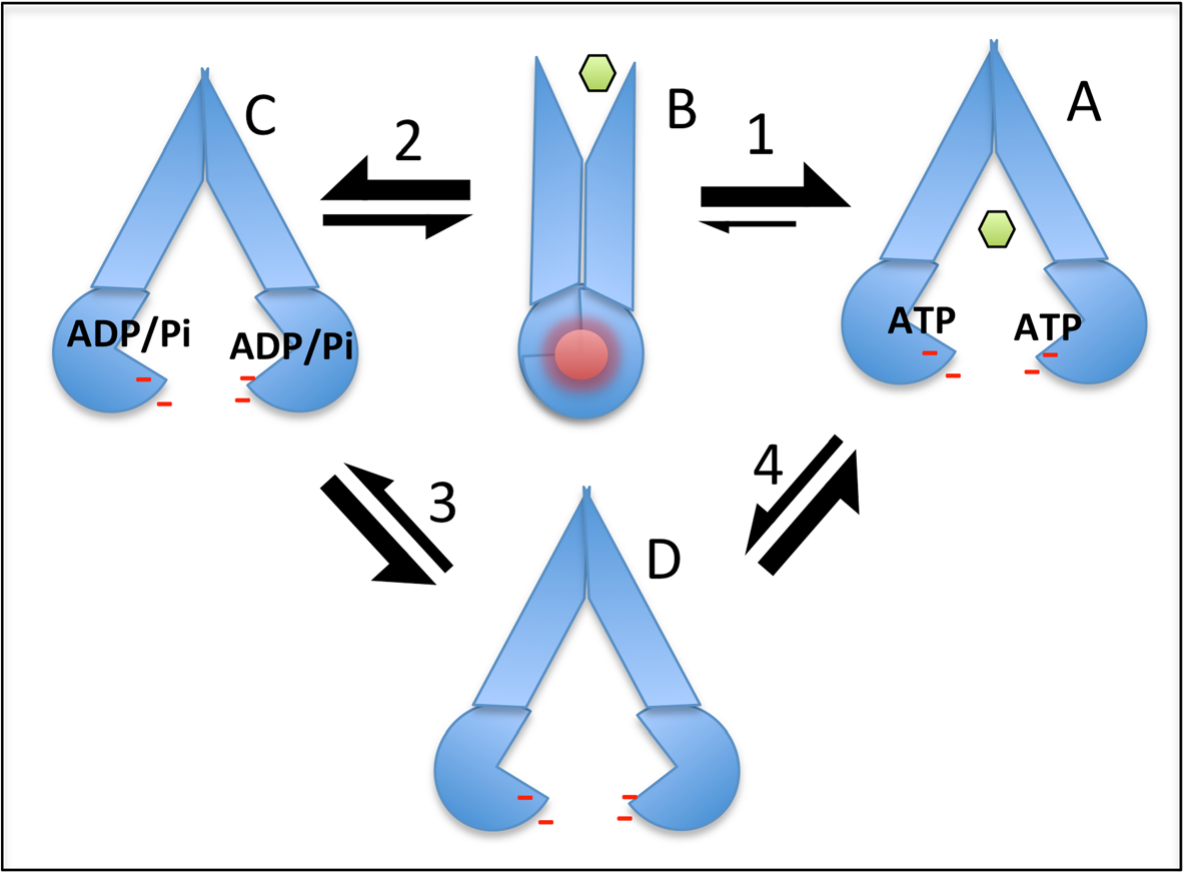
Model of P-glycoprotein action. Under physiological conditions (high ATP, no allocrite) the inward-facing state (**a**) predominates over the outward-facing state (**b**) which is unstable because of bringing together negative charges (red dashes, red circle). Allocrite binding (green hexagon) increases the chances of formation of the outward facing state and export of the allocrite to the outside. Hydrolysis of ATP can occur during the lifetime of the outward-facing state and hence there is a finite chance of forming the post-hydrolytic state (**c**), even in the absence of allocrite. Dissociation of inorganic phosphate (Pi) and ADP results in the formation of the transient apo-state (inward-facing, **d**) before ATP re-binds and returns the system to the starting point (**a**). The thickness of the two-way arrows at stages 1–4 indicate the relative proportions of the different paired states, but are not intended to be quantitative

A simple scheme incorporating the switching between the inward and outward-facing states is shown in Fig. 4: In this model, the inward-facing, ATP-bound conformation of P-gp predominates at the high ATP levels in the cell (equilibrium at stage 1). Formation of the outward-facing state may be dis-favored because of electrostatic repulsion in the NBD dimer (red circle) combined with the need for removal of steric blockers (the NBD1-TMD2 linker region and the polar headgroups of lipids filling the TMD gaps). These various properties of the exporter could prevent unwanted ATP hydrolysis under normal physiological conditions where no allocrite was present. Formation of the outward-facing state in the absence of allocrite is therefore probably a rare event and will determine the basal ATP hydrolysis rate. When a P-gp allocrite is bound (green hexagon), the equilibrium at stage 1 is shifted, and formation of the outward-facing state becomes less rare. Upon formation of the outward-facing state, the escape of allocrite to the outside of the cell and ATPase activity can occur (followed by a rapid reversal to the inward-facing, post-hydrolytic state – stage 2). Inorganic phosphate and ADP must then dissociate to form the apo-state (stage 3) before ATP can re-bind (equilibrium 4). There is no obvious reason why allocrite should not bind to any of the inward-facing states detected so far in structural studies. Access to the two proposed allocrite-binding sites may be via the nearby gaps between transmembrane helices 3&4 and 9&10. If trapped by vanadate, then the protein will remain in the inward-facing state with no further transport, nor ATP binding and hydrolysis. Stabilisation of the outward-facing state may only be possible by mutation of certain residues at the NBD interface (such as the Walker B motif Glutamate residues) or by cross-linking the NBDs when they are transiently together.

Whilst satisfying most of the structural and biophysical data on purified P-glycoprotein, the model in Fig. 4 does not concur with the more generally-accepted idea that *ATP binding* to P-glycoprotein and similar ABC exporters *drives* dimerization of the NBDs and the switch to the outward-facing state with concomintant export [26, 27]. One might reject the implications of the published structural and biophysical studies because they represent isolated systems. The importance of the lipid bilayer, for example, may be crucial [28–31]. Secondly, it is possible that the phosphorylation state of the NBD1-TMD2 linker region may be involved in regulating the switch from inward- to outward-facing states [32], as implied for CFTR/ABCC7 [20]. However if one changes the word ‘*drives*’ to the four words ‘*increases the chance of* ’ in the first sentence of this paragraph, then the models for P-glycoprotein mechanism become more compatible. The model derived from the structural and biophysical data would propose the need for the combined presence of ATP *and* allocrite in P-glycoprotein to sufficiently favour the formation of a (still rare) outward-facing state. Depending on the allocrite, one observes increases in ATPase activity by P-glycoprotein of 2–16 fold [11, 30, 33], which implies that there is ample capacity to readjust the imbalance between inward- and outward-facing states in the system. Studies of P-gp under cellular conditions using a conformation-specific monoclonal antibody (UIC2) imply that wild-type human P-gp exists in a mixture of inward and outward-facing conformations, but favours the inward-facing state under the normal cellular environment [34].

## Methods

### Materials

Yeast growth media was purchased from Formedium (Hunstanton, Norfolk, UK). Reagents for protein purification, sodium orthovanadate and disodium ATP were from Sigma-Aldrich (Dorset, UK). n-Dodecyl-β-Maltoside detergent (DDM) was from Merck Chemicals (Nottingham, UK). HisTrap HP and Superose 6 columns were from GE Healthcare (Buckinghamshire, UK). Quantifoil R 1.2/1.3 grid was purchased from Agar Scientific (Essex, UK).

### Mouse P-gp expression in *Pichia pastoris*

Cells containing the *opti-mdr3* gene used in this study were kindly provided by Prof. Ina L. Urbatsch, Texas Tech University [35]. Cell culture was conducted using a previously described shake-flask method with minor modifications [36]. Protein expression was initiated by an addition of 0.5% (*v*/v) methanol and boosted with an equivalent addition of methanol at 24 and 48 h after the first induction.

### Protein purification

Cell rupture and microsome preparation were described previously [37]. Microsomes were diluted to 2.5 mg/ml prior to solubilisation in detergent-containing buffer (50 mM Tris pH 8.0, 10% (v/v) glycerol, 50 mM NaCl, 1 mM 2-mercaptoethanol and 2% (*w*/*v*) DDM). Protein purification via immobilized metal affinity chromatography (IMAC) and size-exclusion chromatography SEC were previously described [38] with slight modifications as follows: unbound proteins in IMAC were washed twice with buffers containing 20 and 80 mM imidazole while His-tagged murine P-gp was eluted with 200 mM imidazole. Size-exclusion chromatography (SEC) was conducted using a Superose 6 column. Both steps were carried out in the presence of 0.1% (w/v) DDM. Purified protein was concentrated to 10 mg/ml using a 100 kDa cut-off Vivaspin concentrator, flash frozen in liquid nitrogen and stored at − 80 °C.

### Cryo-electron microscopy

P-gp was incubated with 2 mM Na_2_ATP, 2 mM MgCl_2_ and 1 mM Sodium Orthovanadate for 15 min at 37 °C prior to deposition onto grids as described in [14]. Quantifoil 200 or 400 Au grids with a R1.3 spacing pattern where washed multiple times with chloroform in a glass dish on filter paper to remove hydrophobic contaminants residual from the manufacturing process and left to air dry for several minutes. Grids were then placed on a parafilm-wrapped glass slide before glow discharging for 2 min at 25 mA. Samples in vitreous ice were prepared using a FEI Vitrobot MkIV – 3 μl of protein sample at a concentration of 1 mg/ml was gently placed in the middle of the grid before immediately blotting for 4 s and flash freezing in liquid ethane. Grids were assessed for ice thickness and specimen quality using a Polara G30 before shipping to the eBIC UK National facility for high resolution data acquisition on a FEI Titan Krios G2 microscope. Data were recorded at 300 KeV using a 20 mV energy filter and a Gatan K2 electron detector – images were recorded using a total dose of ~ 70 e^−^ over 40 frames at a calibrated sampling increment of 1.06 Å/pixel. Data were recorded using a defocus range between − 1 and − 4 μm sampled at 0.25 μm increments. 2800 movie stacks were recorded and drift was removed and dose weighted using MotionCorr 2 [39].

### Image processing

Images were CTF corrected using gCTF [40] and over 80% of the recorded data had resolution extending between 4 and 7 Å – the remaining images were discarded. A random selection of 30 images was used for initial autopicking reference particles using gAutomatch (developed by Zhang K, MRC Laboratory of Molecular Biology, Cambridge, UK) and a small dataset of 4000 particles was processed to provide picking templates. gAutomatch was then used to pick an initial first pass selection of ~ 580,000 particles. Data was subsequently processed in RELION [41]– following CTF correction as above, and multiple rounds of 2D classification were performed to remove non-P-gp particles. Examination of the 2D classes suggested that P-gp was in an inward- facing conformation. A low-resolution start model was generated from classes representing different Euler angles and then 3D classification subdivided the dataset into three 3D classes with one class demonstrating a higher resolution than the other two. This class was subjected to auto refinement, producing a final 3D map with an estimated ‘gold standard’ resolution assessment of 7.9 Å [42, 43]. The map was post-processed using a mask designed to fit locally around the protein density with a 6 pixel soft fade and automatic b-factor detection. Local resolution in the map was assessed using the Resmap software [17].

### Model building and molecular dynamics flexible fitting

Initially, rigid-body fitting of the 4ksb model into the cryo-EM map was performed using the UCSF Chimera software ‘fit-in-map’ routine [44]. The VMD software [45] was then employed to prepare the system and to generate all the necessary configuration files for the simulation. In a separate system, ADP molecules were added to the fitted 4ksb model (using the available inward-facing structures of ABC transporters with nucleotide bound as a guide). Small adjustments to the Walker A loops in 4ksb were made in order to accept the ADP molecules without steric clashes. The systems were subjected to flexible fitting using the Molecular Dynamics Flexible Fitting (MDFF) package [46]. A scaling factor of 0.3 kcal/mol was applied to the entire model, representing the force applied to the atoms in order to flexibly fit to the cryo-EM map with a density threshold ϕ_*thr*_ of zero (default value, corresponding to the solvent peak). To maintain the integrity of the secondary structure elements and prevent over-fitting, harmonic restraints were also applied. A simulation run was performed using the NAMD 2.12 software [47] and the CHARMM36 force field [48]. The system was subjected to 100,000 steps (100 ps) of energy minimization, followed by 5,000,000 steps (5 ns) of the production run, without symmetry restraints, in a vacuum environment and at a constant temperature of 300 K. For the ADP-containing run, an additional 100,000 steps of energy minimization was added at the start of the run, and this corresponded to a preliminary minimization phase including a different scaling factor of 0.1 kcal/mol to allow the 4ksb model to correctly adapt to the modifications applied.

UCSF Chimera [44] was used to visualize and analyze trajectories and finally to compare cross-correlation coefficients at defined time steps, corresponding to models extracted at different frames (Additional file 1: Figure S5). The MDFF identified significant additional density compared to the fitted model. It was possible that this additional density might influence the MDFF, giving a distorted model. This was checked by doing a simple rigid-body fitting of the first structural half of the molecule (TM1–3,6,10,11,NBD1) followed by the second half (TM7–9,12,4,5,NBD2) for different atomic models. For display, the Chimera routine “Color Zone” was employed to colour regions of the map within 2 Å of the fitted model.

## Additional file

Additional file 1: Additional details on the protein purification; image processing; map resolution and atomic model fitting quality and comparison of the map with prior low resolution data. (PDF 1088 kb)

## Abbreviations

ADP: Adenosine diphosphate
ATP: Adenosine triphosphate
DDM: n-Dodecyl β-D-maltoside
MDFF: Molecular dynamics flexible fitting
NBD: Nucleotide-binding domain
P-gp: P-glycoprotein
TMD: Transmembrane domain
