# Supplementary material for: Novel features in the structure of P-glycoprotein (ABCB1) in the post-hydrolytic state as determined at 7.9 Å resolution
Source: BMC Struct Biol. 2018 Dec 13;18:17. doi: 10.1186/s12900-018-0098-z (PMC6293506; doi:10.1186/s12900-018-0098-z)

Supplementary Figure 1: Protein purification profiles. (a) Coomassie-stained SDS-PAGE of mP-gp purified in the presence of DDM via 2-step purification comprising nickel-affinity chromatography (IMAC) and SEC. (b) SEC chromatogram showing absorbance of eluted material at 280 nm. The protein eluted as a main peak with no high molecular mass aggregates observed. Fractions 12-13 were pooled and used for structural studies. The elution peak of the protein was analysed by in-line multi-angle laser light scattering, giving a mass of 243kDa, roughly equivalent to a mP-gp monomer (with the addition of a DDM micelle).

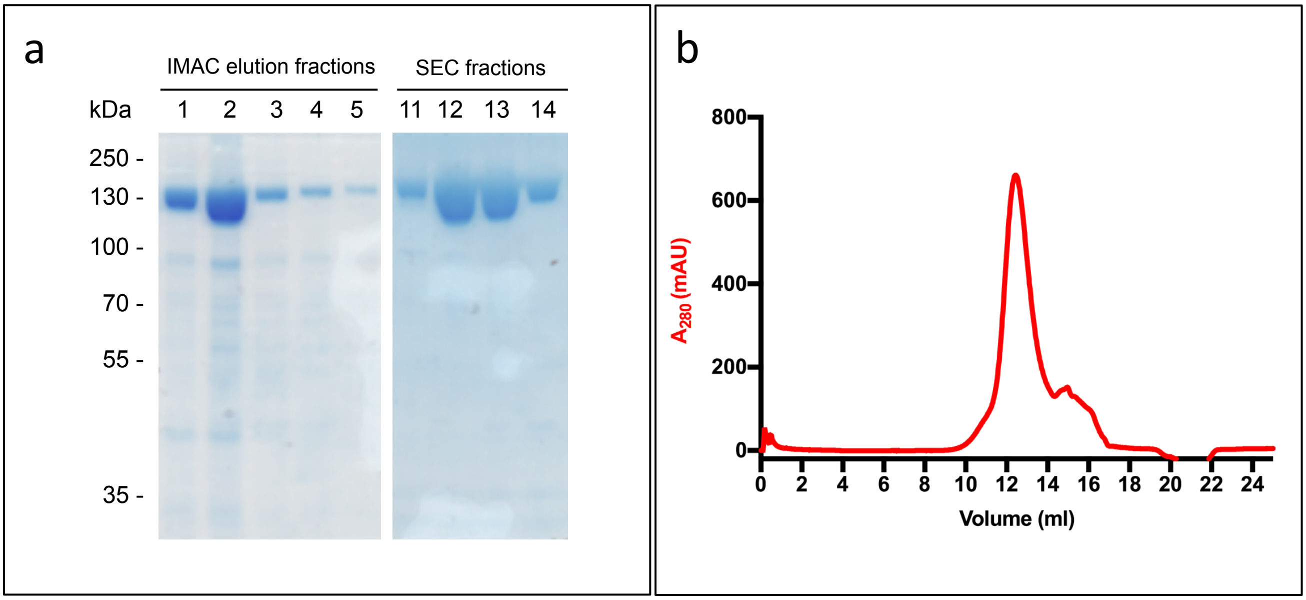

Supplementary Figure 2: Flow chart of the data analysis. 2,209 image stacks were acquired and processed. ~ 550k auto-boxed areas were initially obtained corresponding to P-gp particles as well 'bad' particles (carbon support film, ice, contamination, clumps, etc.). A series of 2D classifications yielded a refined dataset of ~135k good particles with few contaminants. Low-resolution 3D models were generated using selected 2D classes. The particle dataset was re-classified and refined against this to provide a 3D map with high-resolution features. After the resolution limit was calculated, the higher frequency components in the density map were enhanced (map sharpening)

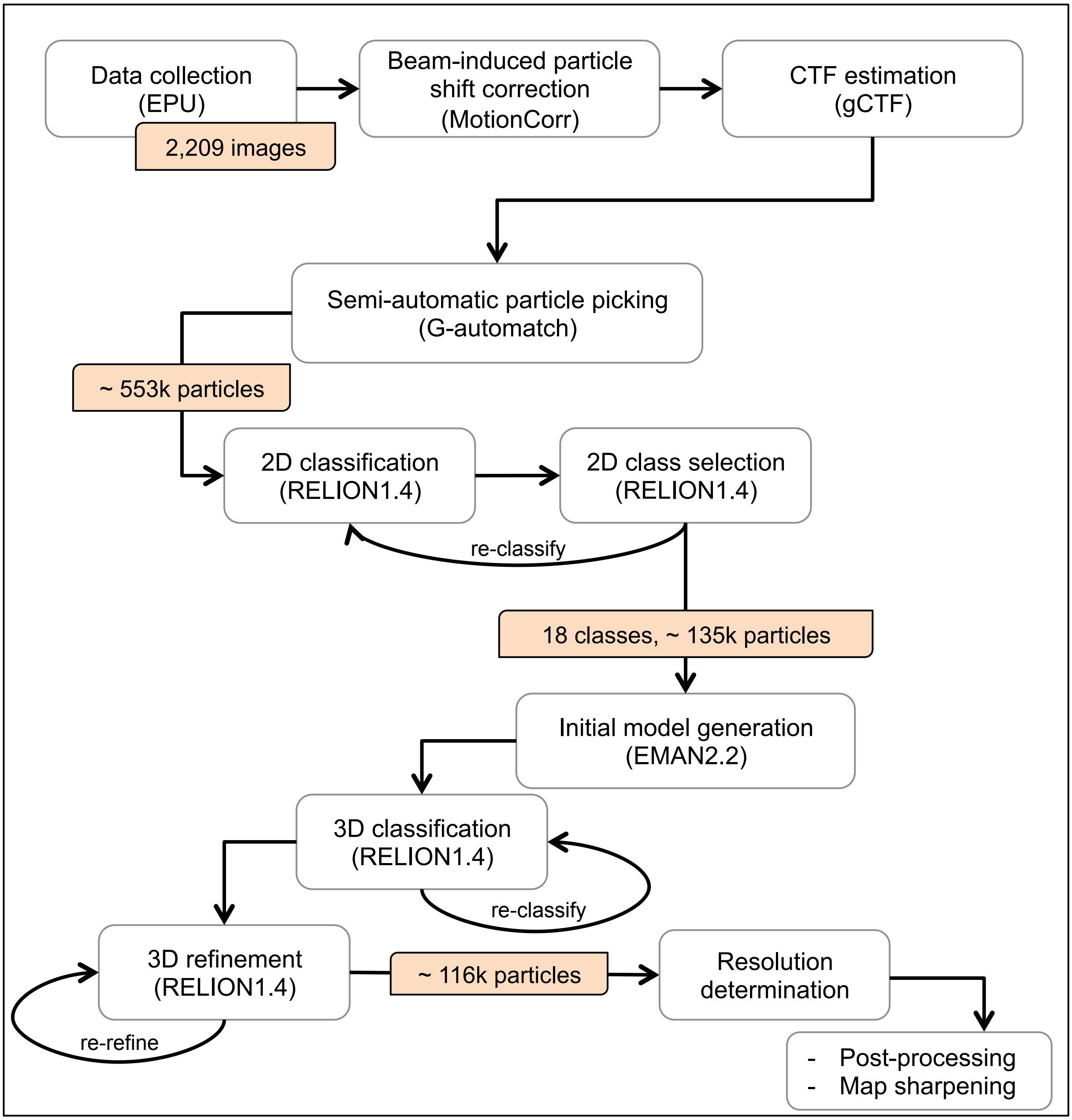

Supplementary Figure 3. (a) Local resolution assessment using the Resmap algorithm and displayed using colours to represent different resolution ranges (colour key is on the right). The detergent micelle shows lower local resolution, as expected. Core protein regions show the highest resolution in the map. (b) Global resolution assessment of the two independently-refined experimental maps using the Fourier shell correlation calculated between them and following the procedure of Chen et al., (25) as implemented in Relion. The green line shows the plot for unmasked maps where surrounding noise contributes more and the blue line shows the plot with soft-masked maps. The red line shows the FSC plot for the data with the phases randomised beyond 10Å resolution. The black line shows the corrected curve (FSC_{true}), yielding a resolution estimate of 7.9Å.

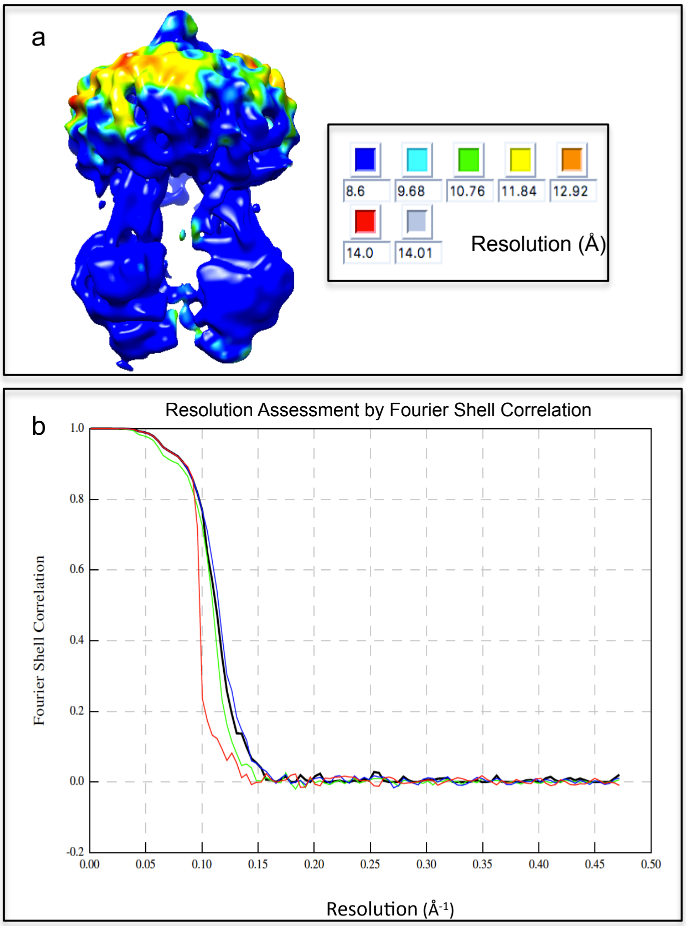

Supplementary Figure 4. Comparison of cryo-EM maps. The upper panel shows mP-gp in the vanadate-trapped state in this study (purple) and in a prior study employing an F_{AB} fragment binding to the extracellular surface of the protein (Frank GA, *et al., Mol Pharmacol* 2016, **90**:35-41 - blue mesh). The separation of the NBDs is similar. The lower panels show the mP-gp map from this study (grey), but before post-processing (i.e. sharpening) and illustrate that the additional densities assigned to the N-terminal extension (left, yellow) and to the C-terminal end of the linker region (right, yellow) can be clearly distinguished from the nearby detergent micelle. In both representations the rear of the map has been slabbed away for clarity.

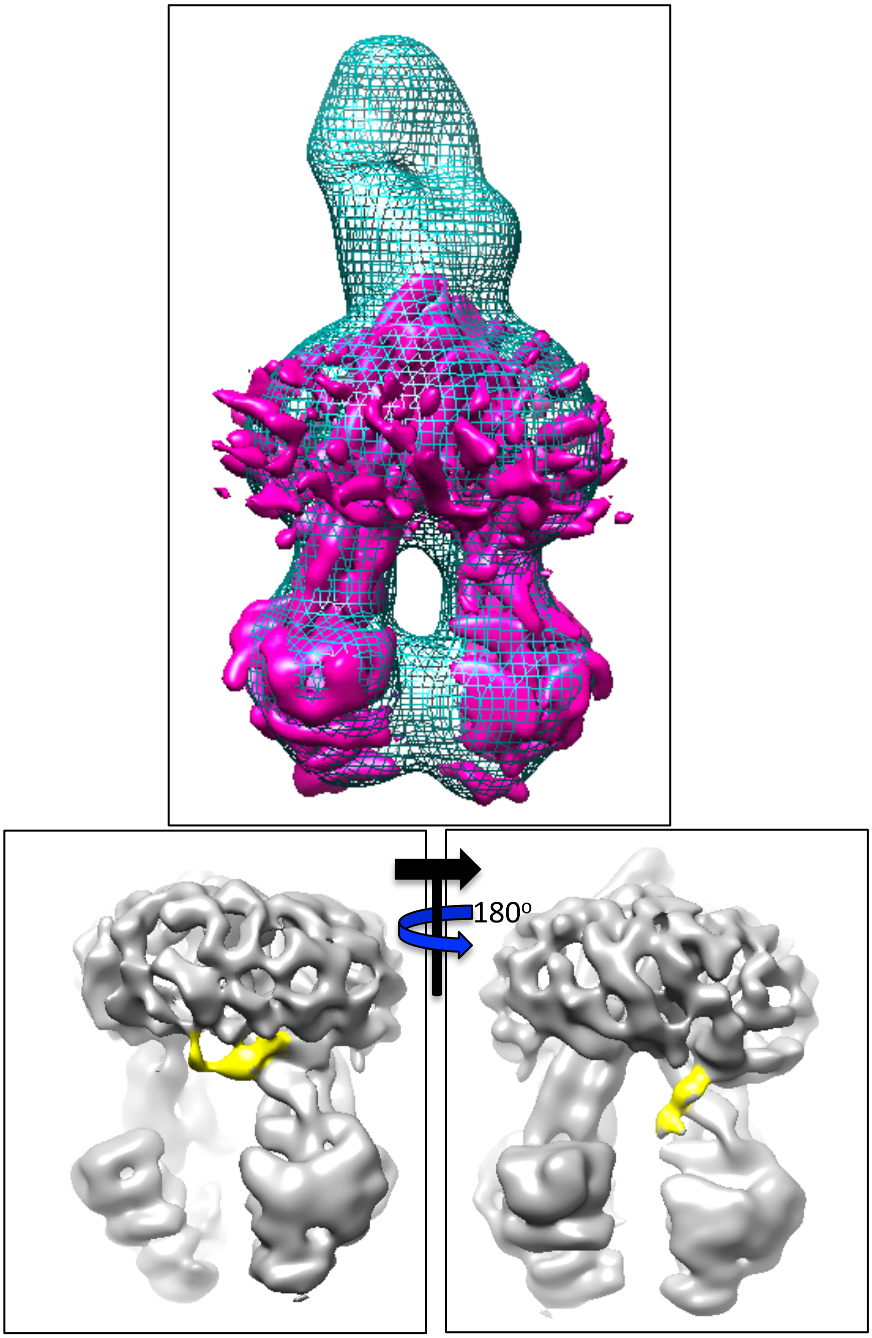

Supplementary Figure 5. Molecular Dynamics Flexible Fitting (MDFF) of the 4ksb atomic model to the experimental map. (a) Root mean squared deviation (RMSD) of the protein backbone with respect to the initial rigid-body fitted model is plotted. (b) Plot of the cross-correlation coefficient (CCC) calculated between the experimental map and a theoretical map calculated from the fitted model at the same resolution as the experimental map. The plots show that after a short adjustment period (panel a), the dynamic fit to the experimental map improves and becomes stable (panel b). Very similar plots were obtained when ADP was included in the MDFF run.

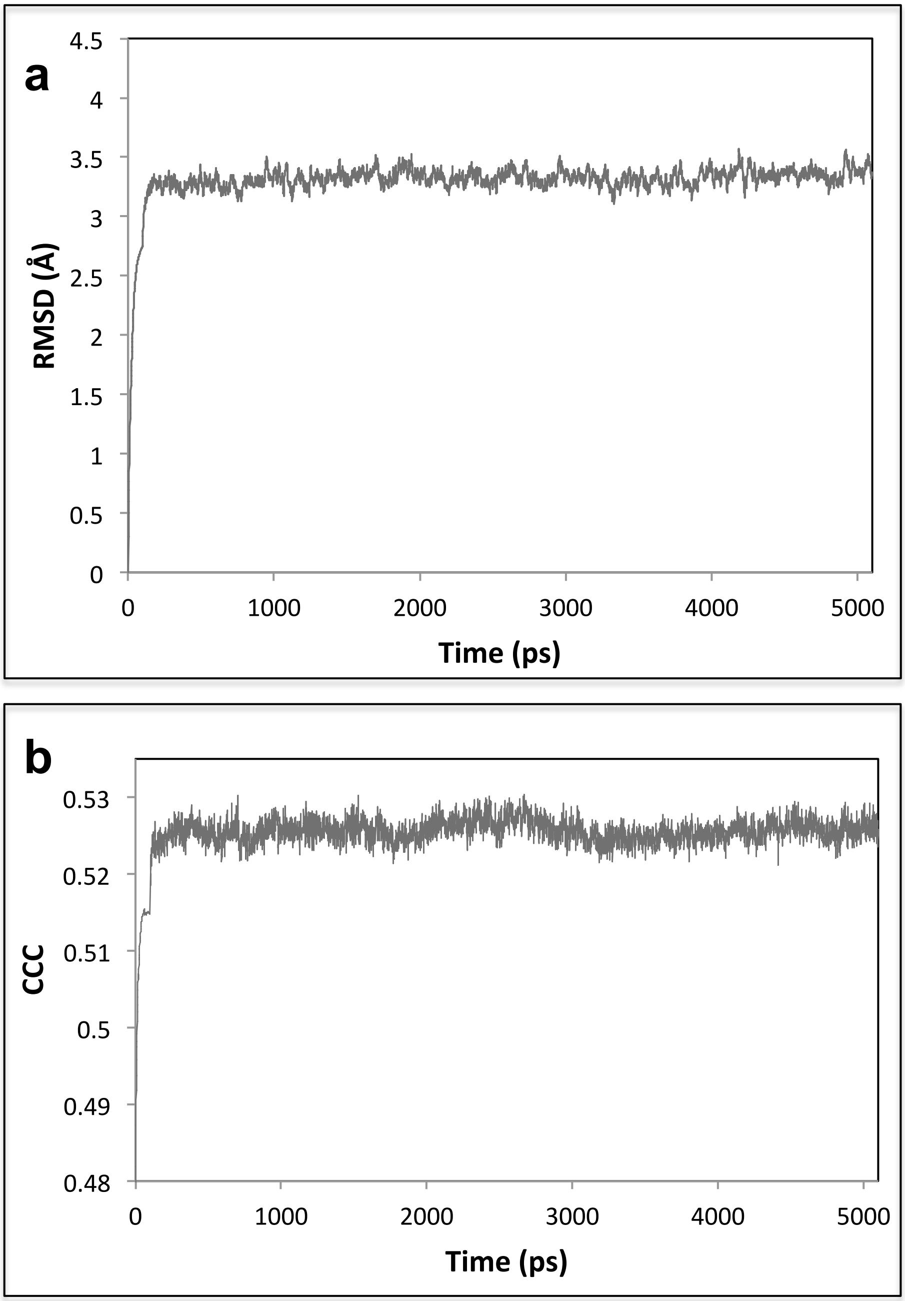

Supplement: Supplementary file 1 — Additional details on the protein purification; image processing; map resolution and atomic model fitting quality and comparison of the map with prior low resolution data. (PDF 1088 kb) [file 12900_2018_98_MOESM1_ESM.pdf]
